# Polysialic acid is upregulated on activated immune cells and negatively regulates anticancer immune activity

**DOI:** 10.3389/fonc.2025.1520948

**Published:** 2025-03-20

**Authors:** Olivia Drummond-Guy, John Daly, Angeline Wu, Natalie Stewart, Katy Milne, Chloe Duff, Brad H. Nelson, Karla C. Williams, Simon Wisnovsky

**Affiliations:** ^1^ Faculty of Pharmaceutical Sciences, University of British Columbia, Vancouver, BC, Canada; ^2^ Deeley Research Centre, British Columbia (BC) Cancer, Victoria, BC, Canada; ^3^ Department of Medical Genetics, University of British Columbia, Vancouver, BC, Canada; ^4^ Department of Biochemistry and Microbiology, University of Victoria, Victoria, BC, Canada

**Keywords:** immune cells, carbohydrates, glycans, macrophages, T-cells, NK cells, B cells, polysialic acid

## Abstract

Suppression of anticancer immune function is a key driver of tumorigenesis. Identifying molecular pathways that inhibit anticancer immunity is critical for developing novel immunotherapeutics. One such molecule that has recently been identified is the carbohydrate polysialic acid (polySia), whose expression is dramatically upregulated on both cancer cells and immune cells in breast cancer patient tissues. The role of polySia in the anticancer immune response, however, remains incompletely understood. In this study, we profile polySia expression on both healthy primary immune cells and on infiltrating immune cells in the tumour microenvironment (TME). These studies reveal polySia expression on multiple immune cell subsets in patient breast tumors. We find that stimulation of primary T-cells and macrophages *in vitro* induces a significant upregulation of polySia expression. We subsequently show that polySia is appended to a range of different carrier proteins within these immune cells. Finally, we find that selective removal of polySia can significantly potentiate killing of breast cancer cells by innate immune cells. These studies implicate polySia as a significant negative regulator of anticancer immunity.

## Introduction

In cancer, immune cell activity is often suppressed by interactions between inhibitory immune signaling receptors and their cognate ligands (1–3). These molecules can be overexpressed both by cancer cells and by other immune cells in the tumor microenvironment (TME) (1–3). Immune checkpoint inhibitors (ICIs) block these interactions and stimulate anticancer immune responses (1–3). Immunotherapies have produced some remarkable results, even in patients with otherwise untreatable disease (4, 5). However, existing immune-based therapies are still ineffective for most patients. For example, less than 40% of patients respond to ICIs even in immunotherapy-sensitive cancers like melanoma (6). This is likely due to immune cells expressing dozens of receptors that may vary in relevance across different patients and cancer subtypes (1–3). As only a few immune checkpoints have approved inhibitors, characterizing new molecules that regulate anticancer immunity is crucial for developing next-generation therapeutics and biomarkers (1–3).

Dysregulated glycosylation has recently come into view as one possible novel axis of immune suppression in cancer. All living cells are coated with a complex matrix of carbohydrate molecules (termed glycans), which can be attached to cell-surface proteins (through both N-linked and O-linked glycosylation) as well as lipids (7). These glycans modulate many aspects of immune recognition. For instance, glycans can directly serve as ligands for immune-regulatory receptors that either enhance or dampen immune cell activity (8, 9). Glycans can also regulate protein-protein interactions involved in immune cell recognition of foreign cell types (10, 11). In cancer, cell-surface glycosylation patterns on tumor cells, stromal cells and infiltrating immune cells can become dramatically altered (7, 9, 12). The upregulation of immune-suppressive glycans can systematically impair the anticancer immune response and mediate resistance to immune checkpoint inhibitors (13, 14). This is seen particularly often for glycan structures that contain sialic acid, a monosaccharide that negatively regulates immune function through a variety of mechanisms (15).

Polysialylation is a highly specific and tightly regulated glycosylation event that generates long glycan chains composed of α2,8-linked sialic acid residues on the terminal sialic acid residues of N- or O-linked glycans (16). The degree of polymerization (DP) of polysialic acid (polySia) can range from 8 to 400 units (17). PolySia is selectively added to glycoproteins by the Golgi localized polysialyltransferases (polySTs) ST8Sia2 or ST8Sia4 (16, 18). The expression of polySia is developmentally regulated and has been well-studied in the context of neural development (16, 18). In healthy adults, polySia is expressed by a limited number of cell types and protein carriers, such as neuronal cells and NK cells (18). The best-described protein carrier of polySia is NCAM1/CD56, which is the dominant carrier of polySia in NK cells. However, other proteins have also been found to be modified with polySia in other tissues (19–21). In a previous study, we detailed polySia expression in primary tumors from breast cancer patients (22). Intriguingly, we detected high levels of polySia expression not only on breast cancer cells but also on tumor-infiltrating lymphocytes (TILs) in the TME (22). High polySia expression on tumor cells was found to associate with poor patient outcomes. A broad sub-population analysis of polySia+ TILs, categorized as either CD56-positive or CD56-negative, revealed polySia-positivity in both groups and linked it to favourable or unfavorable patient outcomes, respectively. These findings indicate that polySia expression is linked to breast cancer progression and may negatively regulate the anticancer activity of CD56-negative tumor-infiltrating immune cells. Characterizing which immune cell subsets express polySia and determining how polySia regulates immune cell function is an important next step.

Several prior reports have described polySia expression in immune cells. Healthy NK cells are known to constitutively express polySia on CD56 (23, 24). Another study found that polySia expression on murine monocyte-derived cells is dynamically regulated by ST8Sia4 and carrier protein expression (25). In the adaptive immune response, human peripheral naïve T-cells are reported to upregulate ST8Sia2 and ST8Sia4 upon activation, which increases polySia expression in a subpopulation of CD4+ T cells (26). However, there has not yet been a systematic study characterizing polySia expression and function in the anticancer immune response. The specific immune cell types that express polySia in the TME remains unexplored. Additionally, it is unclear how polySia is regulated upon immune activation and how polySia may influence anticancer immune function.

In this study, we comprehensively profile expression of polySia on both healthy primary immune cells and on immune cell sub-populations in the TME. PolySia expression is generally low on resting T-cell, B-cell and monocyte populations in healthy donors. However, these cell types all demonstrate expression of polySia in the TME, with macrophage and B-cell populations demonstrating the highest rates of polysialylation. Using primary immune cell activation assays, we then demonstrate that transient stimulation of T-cells and macrophages leads to a distinct increase in polySia expression. In T-cells, we show that much of this polySia expression is present on CD56-negative cells. Using immunoprecipitation assays, we confirm the presence of several novel polysialylated carrier proteins in primary human T-cells. Finally, we use *in vitro* immune killing assays to show that polySia negatively regulates the anticancer effector function of phagocytic macrophages. Taken together, these studies provide key new insights into polySia expression within the immune system and point to a distinct role for polySia in regulating innate anticancer immune activity.

## Results

### Immunophenotyping of both healthy and tumor-associated immune cells reveals dramatic changes in polySia expression associated with tumorigenesis

We first assessed polySia expression on healthy immune cells using PBMCs isolated from a panel of healthy blood donors. We stained these cells with a previously validated polySia-binding antibody, along with an antibody panel against key markers for T-cells (CD3+), NK cells (CD3+/CD56+), monocytes (CD14+) and B-cells (CD19+) (22). These distinct populations were all analyzed for polySia expression using multi-colour flow cytometry. As a control, we enzymatically removed polySia by treating immune cells with Endoneuraminidase-NF (EndoN) prior to polySia immunostaining. EndoN is a bacteriophage enzyme that selectively cleaves 2,8-linked sialic acid chains of DP ≥ 7 (27). This step allowed us to more precisely gate on polySia-positive immune cell populations and alleviates concerns about possible off-target antibody-binding in our flow cytometry assays.

**Figure 1:**
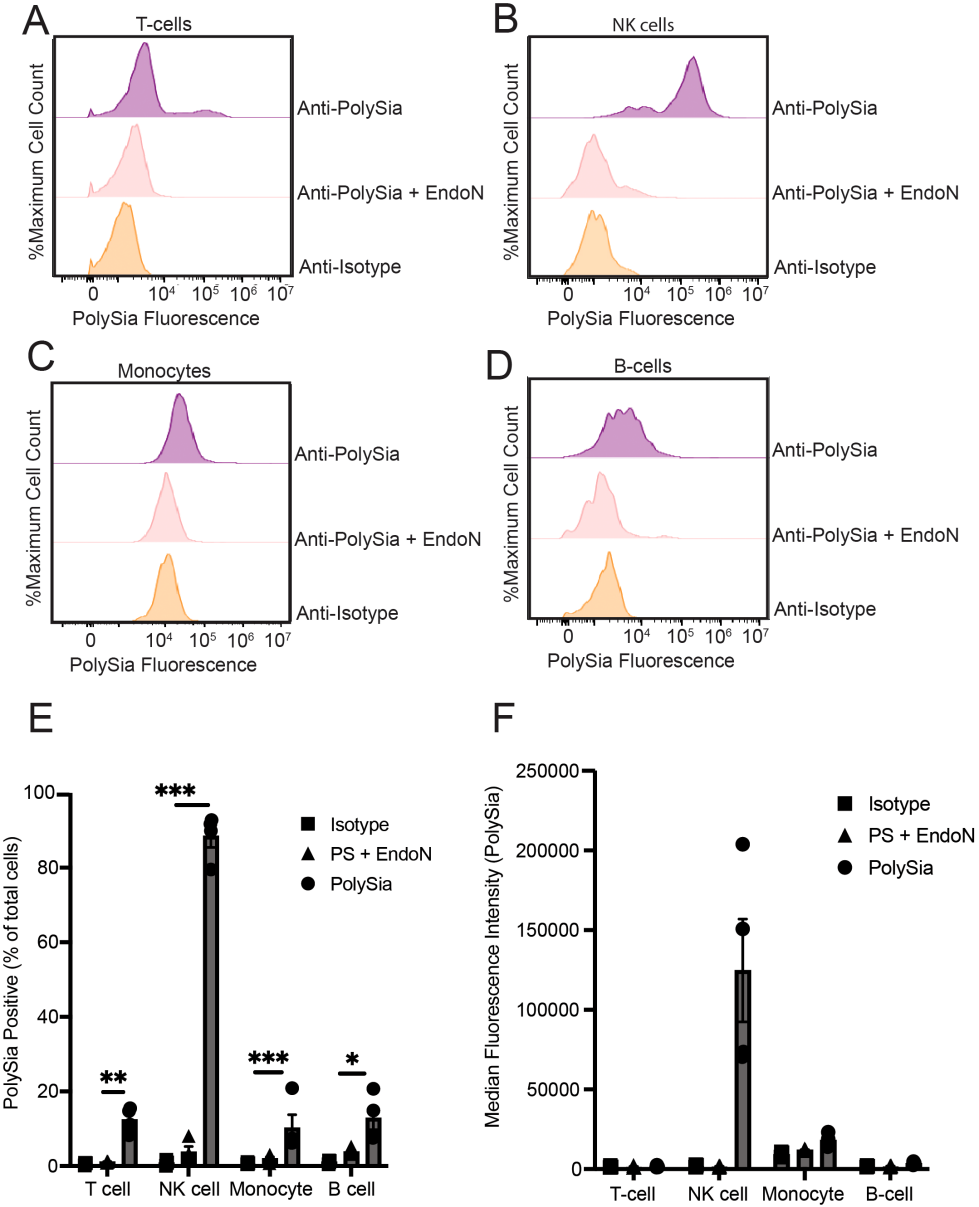
Polysialic acid is selectively expressed on specific subsets of peripheral immune cells. PBMCs were stained with an anti-CD3-PE antibody, an anti-CD14-PE antibody, an anti-CD56-FITC and/or an anti-CD19-PE antibody. PBMCs were also stained with anti-polySia-APC or isotype control. EndoN treatment was also used to remove polySia from the cell surface. Cells were then analyzed using flow cytometry. **(A)** Representative flow cytometry plot of polySia+ naïve T-cells. **(B)** Representative flow cytometry plot of polySia+ NK cells. **(C)** Representative flow cytometry plot of polySia+ monocytes. **(D)** Representative flow cytometry plot of polySia+ B-cells. **(E)** polySia+ % of the indicated immune cell subsets. “PS + EndoN” indicates cells treated with EndoN and stained with an anti-polySia antibody. **(F)** Median fluorescence intensity (MFI) of the indicated immune cell subsets. “PS + EndoN” indicates cells treated with EndoN and stained with an anti-polySia antibody. N=4 donors, error bars indicate SEM. * indicates p<0.05, ** 0.01, *** 0.001.

Our full set of profiling results found that NK cells exhibited strong expression of polySia relative to T-cells, monocytes, and B-cells (**Figures 1A–F**), mirroring previous reports. While expression was minimal or restricted in the other immune subsets, we did note that monocyte and B-cell populations sometimes displayed a small level of staining that was sensitive to EndoN. However, this staining was quite variable and only present in some donors. Within the CD3+ T-cell compartment, we did consistently observe a more distinct sub-population that were polySia positive. We therefore further evaluated the extent of polySia staining within different T-cell subsets. A greater percentage of CD8+ T-cells were positive for polySia expression than CD4+ T-cells (**Supplementary Figure 1A**). Likewise, we observed higher levels of staining in CD45RO+ (memory) cells compared to CD45RO- (naïve) T-cells (**Supplementary Figure 1B**). In all these subsets, polySia+ cells represented a small fraction (<25%) of the total compartment. In some cases, polySia expression also displayed significant donor to donor variability. Taken together, these results confirm that NK cells are the only naïve immune cell population that is predominantly polySia+ in healthy donors. However, they also imply the presence of small sub-populations of polySia+ leukocytes and monocytes. These findings reinforce and extend prior studies of polySia expression patterns within the human immune system (24, 26, 28).

**Figure 2:**
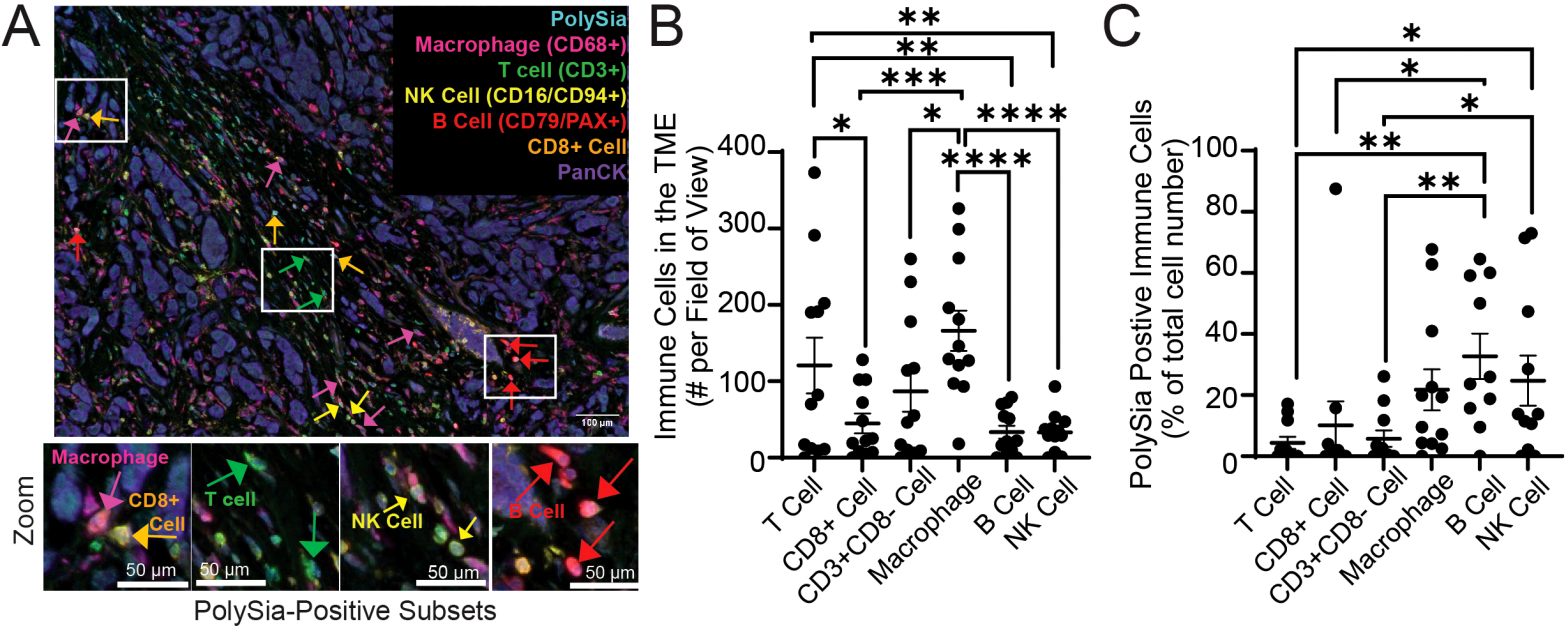
Polysialic acid is expressed on multiple immune subsets in the tumor microenvironment. A breast tumor tissue microarray containing n=12 tumor tissues was stained with antibodies against CD3, CD8, CD68, CD16a, CD94, CD79, PAX5, polySia, and pan-cytokeratin (PanCK). **(A)** A representative image of immune cell subsets and polySia+ cells in the tumor microenvironment is shown. Higher magnification insets (zoom) show polySia-positivity in T-cells (CD3+), CD8+ T-cells, CD3+CD8- T-cells, B-cells (CD79+ and/or PAX5+), macrophages (CD68+), and NK cells (CD16a+ and/or CD94+). **(B)** Analysis of the number of immune cell subsets in each tumor tissue is represented. **(C)** Analysis of the proportion of each immune cell subset positive for polySia. Scale bar = 100 µm and 50 µm (inset). Error bars indicate SEM. p values shown from a one-way ANOVA test: * indicates p<0.05, ** 0.01, *** 0.001, ****p<0.0001.

Immune cells in the TME often exhibit dramatic alterations in polarization and cell-surface marker expression (29). We thus hypothesized that polySia may be significantly upregulated on immune cells in the TME when compared to that of healthy donor PBMCs. We evaluated the expression of polySia on different immune cell subsets in the breast TME using multicolour immunofluorescence (mcIF). Patient tumors were immunostained for T-cells (CD3+), NK cells (CD16a/CD94), macrophages (CD68+), B-cells (CD79a/PAX5), polySia, and pan-cytokeratin (PanCK) (**Figure 2A**). All immune subsets were identified across various breast tumor tissue (n=12), with macrophages being the most abundant infiltrating immune cell (**Figures 2A, B**). Analysis of polySia-positivity in each subset identified varying expression, with NK cells, macrophages, and B-cells demonstrating the highest rates of polysialylation (**Figure 2C**). Taken together with our flow cytometry analysis, these results indicate that polySia expression is dynamically regulated on immune cells and altered in the TME. We therefore sought to better understand how polySia expression is upregulated during immune activation and what role polySia might play in modulating immune function.

### Stimulation and differentiation of peripheral immune cells leads to marked increases in polySia expression on specific immune cell subsets

Immune cells in the TME often exhibit a suppressed, “exhausted” phenotype resulting from chronic stimulation and compensatory upregulation of inhibitory checkpoints (30). We therefore hypothesized that *ex vivo* stimulation of healthy immune cells might induce some of the same changes in polySia expression we observed in the TME. We first tested this hypothesis using healthy primary T-cells. PBMCs from healthy donors were stimulated with anti-CD3 and anti-CD28 antibodies to induce T-cell activation and expansion. To mimic the effects of chronic TCR stimulation, we repeated antibody treatments multiple times over a 14-day stimulation period. This protocol has been found to induce polarization of T-cells in a way that mimics an “exhausted” phenotype (31). We then assessed cells for polySia expression at different time points following stimulation.

**Figure 3:**
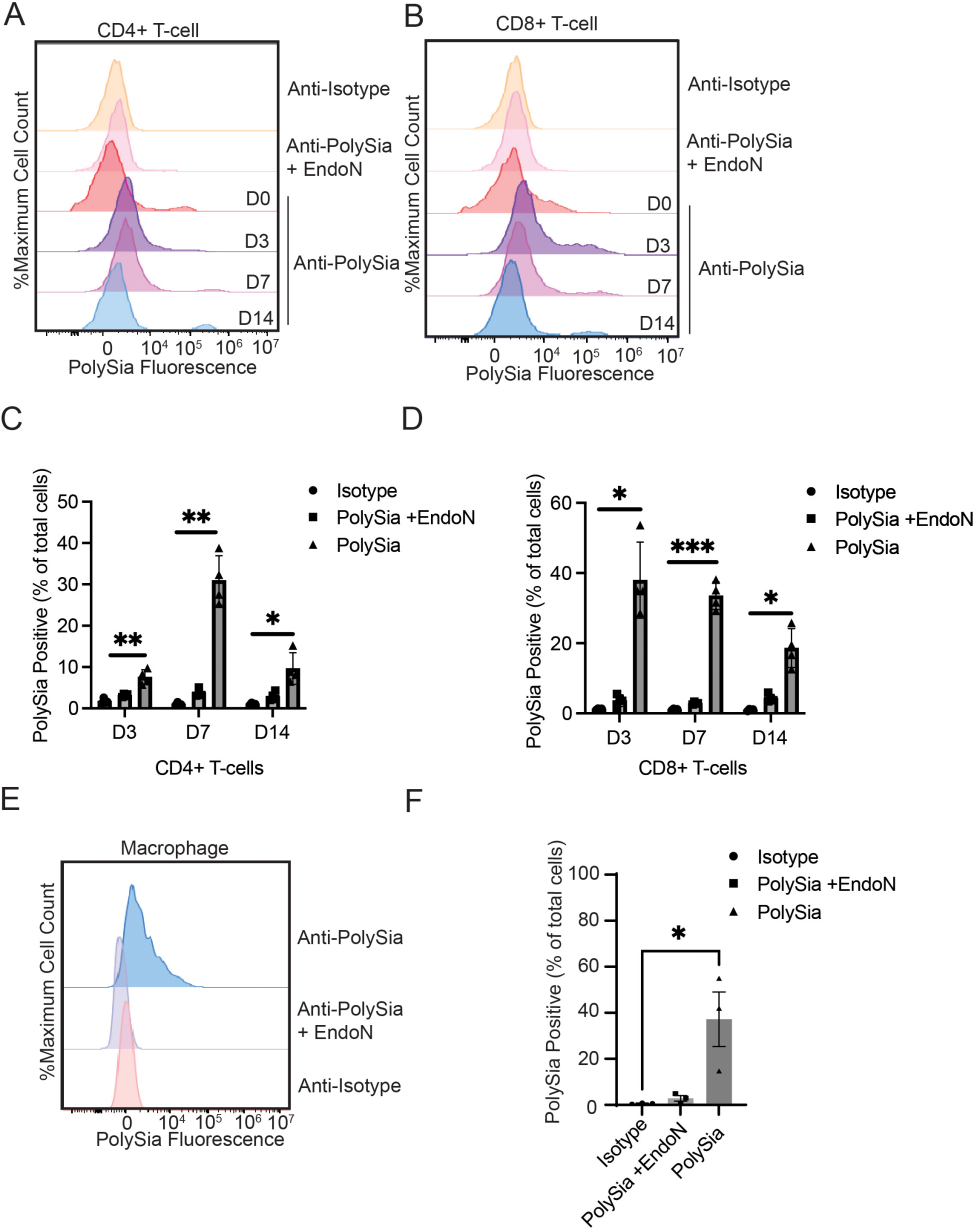
Stimulation of peripheral immune cells selectively increases polysialic acid expression on specific immune cell subsets. T-cells were stimulated with anti-CD3/CD28 antibodies for the indicated times. They were then stained an anti-CD4-bv421 antibody, an anti-CD8a-PE antibody and either an anti-polySia-APC antibody or an isotype control. EndoN treatment was also used to remove polySia from the cell surface. Cells were then analyzed by flow cytometry. **(A)** Representative flow cytometry plot of CD4+ T-cells on day 0, 3, 7 and 14. **(B)** Representative flow cytometry plot of CD8+ T-cells on day 0, 3, 7 and 14. **(C)** The percentage of CD4+ T-cells that were polySia+ at the indicated time points is plotted. **(D)** The percentage of CD8+ T-cells that were polySia+ at the indicated time points is plotted. **(E)** Macrophages were differentiated from primary monocytes for 7 days and stained with either an anti-polySia-APC or an isotype control. EndoN treatment was also used to remove polySia from the cell surface. A representative flow cytometry plot of polySia+ macrophages is shown **(F)** The polySia+ % of total cells is plotted, n = 3 donors. Mean values plotted, error bars indicate SEM. * indicates p<0.05, **p<0.01, *** p<0.001.

Stimulation of T-cells in this manner led to the emergence of a population of polySia+ cells that displayed high levels of antibody staining (100-fold signal over background) (**Figures 3A–D**). In CD4+ T-cells, the polySia+ population was most abundant on Day 7 (30.9 ± 2.9%) and declined after 14 days (**Figures 3A, C**). CD8+ cells displayed different polySia-expression kinetics. PolySia expression was rapidly upregulated on Day 3 with 37.9 ± 5.4% of CD8+ cells expressing polySia (**Figures 3B, D**). Expression remained stable in CD8+ cells over subsequent stimulations. These activation kinetics mirrored those of classical immune checkpoint molecules like PD-1 and LAG-3, which were similarly upregulated by repeated T-cell receptor engagement (32) (**Supplementary Figure 2**). Notably, the shift in polySia staining upon activation was seen only in a fraction of “polySia^Bright^” T-cells. Even at 7 days post-stimulation, the percentage of polySia+ cells did not exceed 50% in either the CD4+ or CD8+ compartment. These experiments thus point to the existence of a polySia+ T-cell population with potentially distinct properties from the overall T-cell pool.

Next, we assessed whether *in vitro*-derived macrophages would exhibit expression of polySia. We isolated monocytes from healthy blood donors and differentiated them into macrophages by treatment with GM-CSF using a previously-established protocol (33, 34). We then evaluated expression of polySia on these cells after 7 days. Macrophages displayed moderate, consistent polySia staining that was sensitive to treatment with EndoN (**Figures 3E, F**). While the overall intensity of polySia expression was lower than that of polySia+ primary T-cells, it was also more consistent, with a greater percentage of cells displaying some level of polySia positivity (**Figure 3F**). Overall, these findings demonstrate that polySia expression in both T-cells and macrophages is regulated by classical activating signaling pathways, suggesting a possible role for this glycan in functional immune regulation.

### PolySia on activated T-cells and macrophages is expressed on non-CD56 carrier proteins

**Figure 4:**
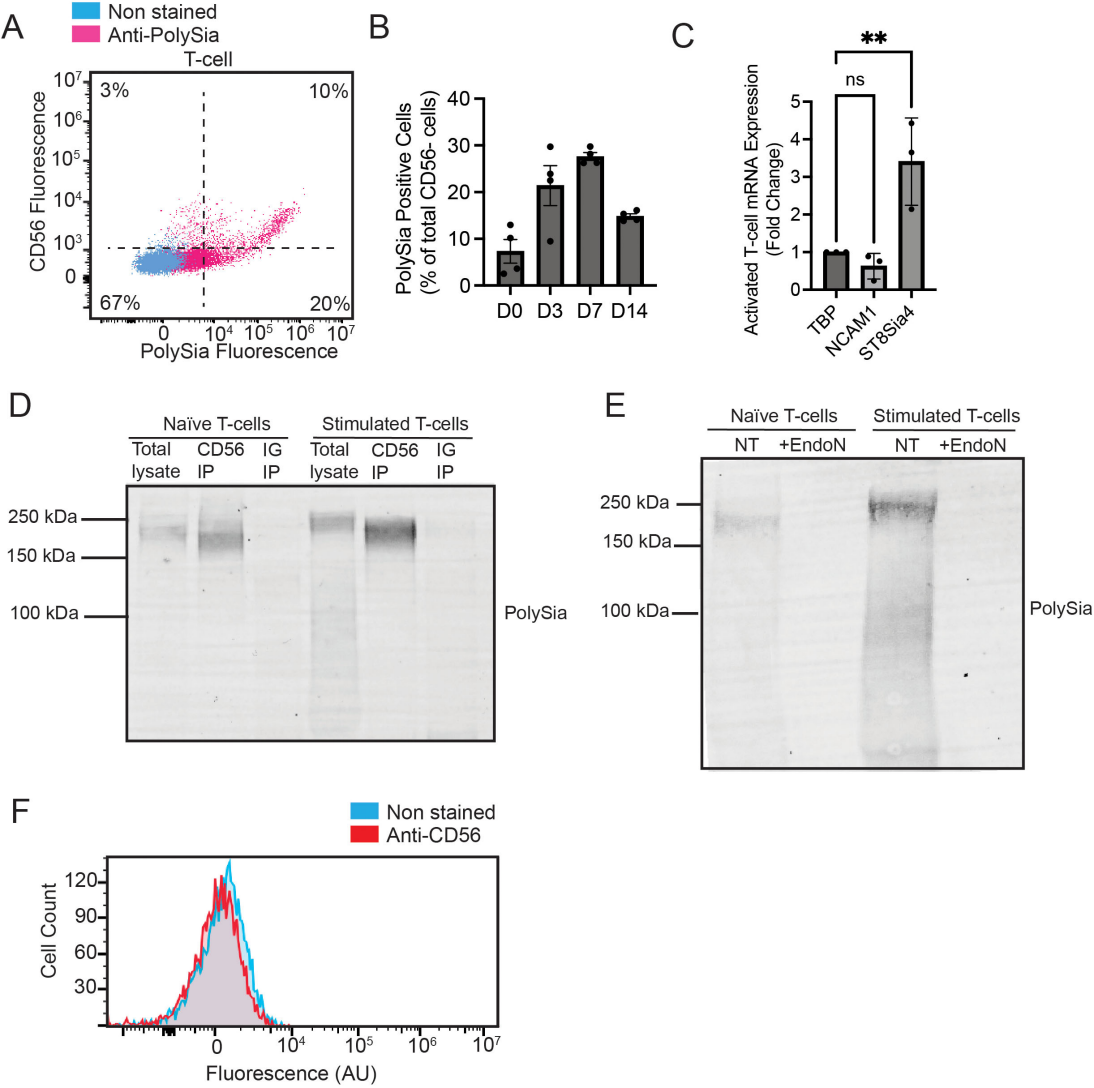
Polysialic acid is expressed on both CD56+ and CD56- activated T-cell subsets. **(A)** T-cells were stimulated as previously described and stained with an anti-CD56-FITC antibody and anti-polySia-APC antibody. Representative flow cytometry plot showing both CD56 and polySia staining is shown. **(B)** T-cells were stimulated for up to 14 days and stained for anti-CD56-FITC antibody and anti-polySia-APC antibody. The %polySia positive cell count of total cells that are CD56- is plotted for different days. **(C)** RNA was extracted from naïve and stimulated T-cells. The mRNA levels of ST8Sia4 and CD56 were calculated relative to the housekeeping gene TBP by qPCR. Mean values plotted, error bars indicate SEM, ** indicates p<0.01. **(D)** Naïve and stimulated T-cell lysates were immunoprecipitated with an anti-CD56 antibody. The bead-binding fraction was then eluted and immunoblotted with an anti-polySia antibody. **(E)** Naïve and stimulated T-cell lysates were immunoprecipitated with an anti-CD56 antibody. The cleared lysate (lacking CD56) was then immunoblotted with an antibody against polySia. Lysates were also treated with EndoN as a control for antibody specificity. **(F)** Macrophages were differentiated for 7 days as previously described and stained with an anti-CD56 antibody. A representative flow cytometry plot is shown. ns, non-significant.

Classically, CD56/NCAM is regarded as the main carrier protein for polySia in the human immune system (24). However, most of this prior work has focused on NK cells. There has been little characterization of the polysialylated proteins on either T-cells or macrophages. To evaluate CD56 expression, we isolated and stimulated primary T-cells, as described above, and analysed polySia and CD56 expression by flow cytometry (**Figures 4A, B**). CD3+ T-cells displayed complex patterns of polySia and CD56 expression. We observed one distinct population that was CD56+ and exhibited extremely high levels of polySia expression. Within this population, CD56 and polySia staining were highly correlated (**Figure 4A**). However, we also observed a second, larger population of T-cells that were negative for CD56 but expressed polySia. The polySia+/CD56- T-cell population was most predominant 7 days after stimulation and declined in relative abundance thereafter (**Figure 4B**). As we have previously observed, distinct populations of CD56+/polySia+ and CD56-/polySia+ positive TILs in tumor tissue, these results strongly imply that polySia is present on distinct cell-surface carrier proteins other than CD56 (22). Increased polySia expression may result from changes in carrier protein (e.g. CD56) or polysialyltransferase (St8Sia2 or ST8Sia4) expression. Evaluation of CD56 and ST8Sia4 expression by qPCR identified a significant increase in ST8Sia4 expression in activated T-cells (**Figure 4C**). CD56 expression, conversely, was unchanged. This demonstrates that polySia expression on T-cells can be regulated by changes in ST8Sia4 expression.

To validate CD56 as the polySia carrier protein, we isolated and stimulated primary T-cells and analysed CD56 for polysialylation by immunoprecipitation. Day 7 stimulated T-cells (previously determined to be the point of maximal polySia expression), where used to isolate and purify CD56. Immunoprecipitated CD56 from T-cell lysates was found to be positive for polySia (**Figure 4D**), demonstrating that CD56 is polysialylated on T-cells. Given our flow cytometry results (**Figures 4A, B**), we also sought to confirm the presence of additional polysialylated protein(s). For this, we analysed cell lysates that had been “pre-cleared” of CD56 through IP and blotted these for the presence of polySia. Lysates were treated *in situ* with EndoN as a control for antibody specificity. This revealed several putative non-CD56 polysialylated proteins in T-cell lysates. Multiple EndoN-sensitive bands were visible, with the most prominent being unknown proteins at ~250 kDa and ~100 kDa (**Figure 4E**).

We performed a similar set of experiments to evaluate whether CD56 carries polySia on primary macrophages. Macrophages were differentiated as above and co-stained with anti-polySia and anti-CD56 antibodies. Here, the vast majority of polySia+ macrophages were CD56- (**Figure 4F**), indicating that macrophages also polysialylate distinct carrier protein(s). Taken together, these results conclusively demonstrate that activated T-cells and macrophages append polySia to multiple non-canonical carrier proteins. Further studies to biochemically purify and identify these proteins may provide important insights into how polySia may regulate immune cell activity.

### PolySia regulates phagocytosis of breast cancer cells by primary macrophages

Finally, we wanted to assess whether polySia might regulate anticancer immune function. In other contexts, polySia has been shown to directly bind to growth factors in ways that modulate cell signaling and differentiation (35). As a highly negatively charged glycopolymer, polySia also plays a role in regulating cell-cell contacts and immune synapse formation (36). We therefore hypothesized that removal of polySia may alter the capacity of immune cells to detect and destroy cancer cell targets. Consistent with the literature, in the breast TME, we identified infiltrating macrophages as a predominate immune cell subset (37). These infiltrating macrophages exhibited robust polySia expression (**Figure 2**). Infiltrating macrophages are known to promote anticancer immunity through phagocytosis of cancer cells and subsequent presentation of tumor antigens (38). We therefore assessed whether removal of macrophage polySia may promote phagocytosis of cancer cells.

**Figure 5:**
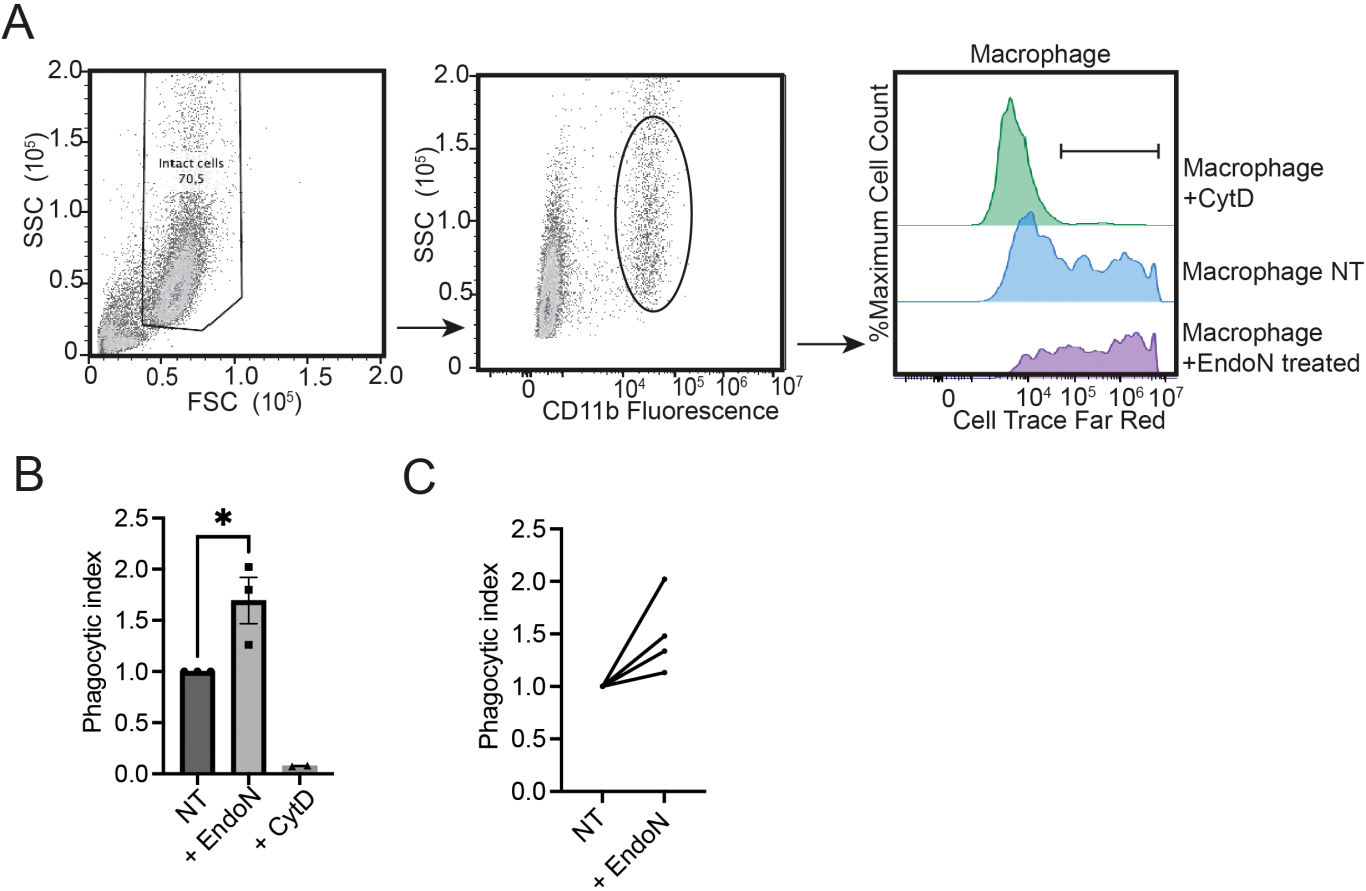
Removal of polysialic acid potentiates the anticancer effector functions of primary macrophages. **(A)** Representative strategy for the analysis of phagocytosis. Monocytes were isolated from PBMCs and differentiated into macrophages over 7 days. CTFR fluorescence was analyzed in the intact CD11b+ macrophage population as shown. The median fluorescence intensity (MFI) of these populations were normalized to generate a phagocytic index. **(B)** Macrophages were derived as in **(A)** and treated with EndoN, the phagocytosis inhibitor CytD (Cytochalasin D) or vehicle control. Phagocytosis index was then determined as above. Plot indicates n=3 independent replicates for a single macrophage donor. Mean values plotted, error bars indicate SEM. * indicates p<0.05 by student’s two-tailed t-test. **(C)** Phagocytosis was assessed as in **(B)** Plot indicates independent replicates for n=4 different macrophage donors.

We differentiated macrophages and measured phagocytosis using the target breast cancer cell line MDA-MB-231. Breast cancer cells were labeled with a fluorescent dye and co-cultured with macrophages for 4 hours. Phagocytosis was then quantitated by measuring the increase in fluorescence in CD11b+ cells (macrophages) using flow cytometry (**Figure 5A**) (34). The phagocytic capacity of macrophages was significantly increased upon removal of polySia with EndoN (**Figures 5A–C**). This trend was observed for n=4 PBMC donors. Tumor antigen presentation, as measured by expression of MHC-II, was unchanged in EndoN-treated macrophages (**Supplementary Figure 3**) These data indicate that polySia negatively regulates initiation of cancer cell phagocytosis by innate immune cells. This finding has important implications for understanding the role of polySia in cancer progression and in the long-term design of polySia-targeted therapies.

## Discussion

Prior work in the field has described polySia expression on various types of primary immune cells, but there has been little exploration of polySia expression on immune subsets in the context of cancer. In this study, we present the first comprehensive characterization of polySia expression on immune cell subsets within the breast tumor microenvironment. By comparing these expression patterns to those of healthy immune cells, we reveal that TILs undergo dramatic remodeling of cell-surface polysialylation in response to tumorigenesis. These changes can be mimicked *in vitro* by stimulation and differentiation of naïve immune cells into activated effector cells. These experiments uncover several important avenues for future study. Most notably, we find novel, distinct sub-populations of CD8+ T-cells that are distinguished by elevated polySia expression and/or expression of the carrier protein CD56. The functional characteristics and physiological role of these polySia+ T-cells remain unclear. In future work, deeper immunophenotyping of polySia+ T-cells may provide further insight into the precise nature of this immune cell subset. Developing methods for isolation of these populations may also help to explore the functional effector properties of these cell types. Another notable discovery arising from our work is the presence of polySia+ B-cells in the TME. PolySia expression was low in B-cells isolated from healthy donors, whereas B-cells were consistently positive for polySia in breast tumors. This is the first report of polySia+ B-cells in any context and should be explored further.

Previous studies have found that polysialylation can occur on a limited number of immune-restricted carrier proteins such as CD56 (39), NRP2 and ESL-1 (40), and CCR7 (28, 41). Polysialylated CCR7 on dendritic cells mediates binding to CCL21 and regulates homing to the lymph node (42). Polysialylated NPR2 and ESL-1 have been documented on monocytes; specifically, microglia and macrophages (40, 43). In microglia, polySia has been shown to regulate inflammatory activation in response to LPS-induced treatment (44). These studies have highlighted the role of polySia in modulating the immune response. Characterizing the full set of polySia+ immune subsets and their carrier proteins can thus help us better understand how polySia regulates immunity. Our work makes a significant advance in this area by showing that polySia is expressed on a wide range of distinct carrier proteins in T-cells, not just the known carrier CD56. In line with other studies (25, 26, 45), our work demonstrates that ST8Sia4 expression is dynamically regulated and increased in activated immune cells. Activation resulted in the emergence of a polySia+/CD56- population. While our study did not identify the polySia carrier protein(s) on T-cells, it is possible that expression of these proteins may also be dynamically regulated. Identifying these specific carrier proteins, determining their function, and evaluating their prognostic or predictive power will be a key challenge for future studies.

The final major finding of our study is that removal of polySia can potentiate killing of cancer cells by phagocytic macrophages. This result agrees well with prior findings showing that polySia negatively regulates phagocytosis of bacterial cells by murine macrophages (25). The mechanism mediating this effect is unclear. Other studies have found that dense, anionic glycosylation (“glycocalyx bulk”) can significantly impair the formation of synapses between phagocytic cells and some cancer cell types (10, 11). PolySia is a bulky, negatively charged glycan and may thus play a similar role. These results could help explain the role of polySia expression during tumorigenesis. In this model, inhibition of phagocytosis would be expected to both directly suppress cancer cell clearance while also inhibiting antigen presentation and the subsequent adaptative anticancer immune response. Our study thus adds to the limited literature describing the negative regulation of polySia in immune cell function. Given this, it is likely that polySia also influences T-cell and B-cell function to control the activation of an immune response. While this function may be biologically important in a healthy state by regulating a controlled immune response, in the context of cancer elevated polySia may represent a blockade to immune activation.

In future, a full description of these mechanisms will require the development of syngeneic *in vivo* tumor models that allow perturbation of polySia expression in the context of a fully intact immune system. These models must be carefully designed, as patterns of polySia expression have been found to differ significantly between human and mouse immune cells (28). Methods for selectively ablating polySia expression on immune cells *in vivo* will also need to be developed. In recent years, numerous new therapeutic strategies have been developed to target cell-surface glycosylation for cancer immunotherapy. These variously include antibody blockade of glycan-binding immune receptors (46) and glycosylated cancer cell ligands (33); enzymatic removal of cell-surface glycans with antibody-enzyme conjugates (47); and inhibition of glycan-producing biosynthetic enzymes (48). Any of these approaches could be applied to selectively target polySia for anticancer therapy. These initial studies thus provide a roadmap for future characterization and therapeutic targeting of polySia in cancer.

## Materials and methods

### Antibodies & reagents

The following list details the full set of antibodies that were used for western blot, flow cytometry profiling studies and immunofluorescence staining.

### PBMC isolation

Anonymous, healthy blood donor samples were sourced from either STEMCELL Technologies or Canadian Blood Services. Leukoreduction system (LRS) cones/buffy coats were processed within 24 hours of the initial blood draw. 8-10 mL (for LRS cones) or 40 mL (for buffy coats) of healthy donor blood was diluted to 35 mL or 70 mL, respectively, in PBS, then layered gradually onto 15 mL Ficol-Paque (GE Healthcare). Samples were centrifuged at 1100xG in a table-top, swinging bucket centrifuge with the brake off (minimum acceleration/deceleration) for 20 minutes at room temperature. PBMCs were carefully isolated from the Ficol/PBS interface using a 1ml pipette, then diluted directly to 50 mL using PBS before being centrifuged at 600xG for 10 mins (acceleration 9, deceleration 9). Cells were resuspended in 50 mL PBS, counted and cells were centrifuged again for 10 minutes at 600xG (acceleration 9, deceleration 9). 50x10^6^ per vial of PBMCs were then frozen in FBS containing 10% DMSO overnight at -80°C in a cryogenic freezing chamber. PBMCs were then transferred to a liquid nitrogen storage unit for long term storage.

### Multicolour immunofluorescence staining and analysis

Breast cancer tissue microarrays (TMAs) were obtained from US Biomax (Rockville, MD, USA). A panel containing antibodies against CD3, CD8, CD16a, CD94, CD68, CD79a, PAX5, pan-cytokeratin, and polySia, was optimized and employed for the multicolor IF. TMA slides were deparaffinized in xylene followed by graded alcohols, fixed for an additional 20 min in neutral buffered formalin and rinsed with dH2O. Antigen retrieval was first performed in a Decloaking Chamber Plus with Diva Decloaker (all products from Biocare Medical unless otherwise indicated). Eight rounds of staining were employed whereby each antibody was added in succession. Details of the primary and secondary antibodies used are listed in the ‘Antibodies’ section. Nuclei stained with DAPI and Prolong Diamond Antifade Mountant (Thermo-Fisher) was added and TMAs were coverslipped. Details of the primary and secondary antibodies used are listed in **Table 1**. TMA slides were scanned using the Vectra Polaris multispectral imaging system (Akoya) and.im3 image cubes were generated (following the manufacturer’s instructions) for downstream analysis. InForm image analysis software (v2.4.4; Akoya) was used to analyze the spectra for all fluorophores. Cells were phenotyped as positive or negative for each of the eight markers in the panel. The study is compliant with all relevant ethical regulations on the use of human tissue and study approval was obtained from the Institutional Ethics Review Board of UBC (IRB#H17-01442).

**Table 1:** Antibodies used in immunofluorescence studies.

1^0^ Ab	Company	Cat#	Clone	Dilution	2’ Fluorophore	Dilution
Polysialic acid	Absolute Antibody	Ab00240-2.0	735	1/200	570	1/200
CD16a/CD94	Abcam	ab183354/ab235441	SP175/EPR21003	1/850 + 1/4000	520	1/500
CD3	Biocare	CD110	PS1	1/400	480	1/200
CD68	Abcam	Ab192847	SP251	1/300	650	1/500
CD8	Cell Marque	108M-95	C8/144b	1/500	620	1/200
CD79a/PAX5	Cell Signalling/Abcam	13333/ab109443	D1X5C/EPR3730	1/250 + 1/1000	690	1/200
PanCK	Biocare	CM162	AE1/AE3 + 5D3	1/300	TSA-DIG	1/100

### Cell culture

MDA-MB-231 cells were sourced from the American Type Culture Collection (ATCC, Cat. no.: HTB-26) and cultured in Dulbecco’s Modified Eagle Medium (DMEM, Cat. No.: 319-005-CL, Wisent Bioproducts) medium supplemented with 10% Fetal Bovine Serum (FBS, Thermo Fisher Scientific, Cat. No.: 12483-020) at 5% CO2 and 37°C. Following isolation, PBMCs were cultured in either Roswell Park Memorial Institute (RPMI, Gibco) with 10% FBS or in differentiation medium specific to a given cell type (detailed below).

### T-cell activation and expansion

PBMCs isolated from healthy donor supplied leukocyte reduction system (LRS) cones (Canada Blood Services & STEMCELL) were plated in a 10cm plate in ImmunoCult™-XF T Cell Expansion Medium (STEMCELL Technologies) overnight to isolate T-cells. The next day, T-cells were treated with ImmunoCult™-XF T Cell Expansion Medium (STEMCELL Technologies) supplemented with 1μg/ml CD3, 1μg/ml CD28 and 0.1ng/ml IL-2 (PeproTech, Cat. No.: 200-02-10UG) and re-stimulated every 2-3 days using the same conditions.

### Macrophage generation

PBMCs were isolated from healthy donor supplied leukocyte reduction system (LRS) cones (Canadian Blood Services) as described previously. 500k PBMCs per well were plated in a 96-well flat-bottom plate in Iscove’s Modified Dulbecco’s Medium (IMDM, Corning) supplemented with 10% human ab serum (Gemini). Macrophages were differentiated into M1 phenotype by supplementing the media with 100ng/ml lipopolysaccharide (LPS) at day 4 and were cultured until use at day 7-9.

### Flow cytometry

To determine polySia expression, naïve or activated immune cell subsets were plated in a V-bottom 96-well plate, centrifuged at 500xG for 5 minutes, and washed with PBS. The cells were stained with anti-PSA, isotype control anti-mouse IgG or other antibodies listed above, in PBS for 30 minutes on ice in the dark. After the incubation, cells were centrifuged at 500xG for 5 minutes, washed with PBS, centrifuged at 500xG for 5 minutes and resuspended in PBS. All measurements obtained by flow cytometry using the flow cytometer (Becton Dickinson) and analyzed using FlowJo (version 10.8.2) software (Becton Dickinson). Events analysed were single, viable cells as dead cells and doublets were excluded using FSC-A vs SSC-A and FSC-A vs FSC-H gating, respectively. The primary threshold (trigger level) was set to automatic and the flow rate was 60 µL/min. For profiling PBMCs, the gain was set to: FSC: 101, SSC: 178, FITC: 145, APC 1408, PE: 988. A total of 30k events were collected. For profiling T-cells, the gain was set to: FSC: 79, SSC: 105, FITC: 40, APC: 1478, BV421: 81, PE:111. A total of 30k events were collected. For profiling macrophages, the gain was set to: FSC: 53, SSC: 21, APC: 131, PE: 27. A total of 10k events were collected. At minimum, 3000 events in the APC+ gate were collected. Antibodies used for flow cytometry studies are listed in **Table 2**.

**Table 2:** Antibodies used in flow cytometry studies.

Antibody	Company	Catalog No.	Application	Concentration
Anti-polysialic acid [clone 735]	Absolute Antibody Ltd.	ab00240-2.0	Western blot	1:500
Anti-NCAM1/CD56 [clone 123c3]	Santa Cruz Biotechnology	sc-7326	Western blot, immunoprecipitation	1µg ab to 1mg of lysate protein
Isotype IgG anti-mouse	Santa Cruz Biotechnology	sc-136967	Western blot, immunoprecipitation	1µg ab to 1mg of lysate protein
IRDye^®^ 800CW Goat anti-Mouse	Li-Cor	926-68070	Western blot	1:10 000
Anti-polysialic acid-APC [clone 735]	Absolute Antibody Ltd.	ab00240-2.0	Western blot	1ng/ml
IG-APC	Invitrogen	17-4724-81	Flow cytometry	1ng/ml
CD3-FITC	Biolegend	300306	Flow cytometry	1:200
CD3-PE	Biolegend	300408	Flow cytometry	1:200
CD8a-PE	Biolegend	301007	Flow cytometry	1:200
CD4-PE	Biolegend	317409	Flow cytometry	1:200
CD4-APC	Biolegend	300514	Flow cytometry	1:200
CD4-BV421	Biolegend	317433	Flow cytometry	1:200
CD45ro-BV421	Biolegend	304216	Flow cytometry	1:200
CD19-PE	Biolegend	302208	Flow cytometry	1:200
CD56-FITC	Biolegend	304603	Flow cytometry	1:200
CD14-PE	Biolegend	301805	Flow cytometry	1:200
CD11b-PE	Biolegend	101208	Flow cytometry	1:200
PD1-436	Invitrogen	62-2799-42	Flow cytometry	1.5:50
LAG3-421	Invitrogen	404-223-41	Flow cytometry	1:50
IG-421	Invitrogen	404-4714-81	Flow cytometry	0.625:100
IG-436	Invitrogen	62-4714-80	Flow cytometry	1:50
HLA-DR	Biolegend	365605	Flow cytometry	1:200
Cell Trace Far Red	Invitrogen	C34564	Cell stain for killing assay	4µM
CD3	Bio X Cell	BE0001-2	Stimulation T-cells	1µg/ml
CD28	Bio X Cell	BE0291	Stimulation T-cells	1µg/ml

### Immunoprecipitation

T-cells isolated from healthy donor supplied leukocyte reduction system (LRS) cones (Canadian Blood Services) were plated in a 10cm plate in ImmunoCult™-XF T Cell Expansion Medium (STEMCELL Technologies) overnight stimulated as per described above. Cells were lysed in cold RIPA lysis buffer with 1x Halt Protease Inhibitor (Thermo Fisher Scientific, Cat. No.: 1862209), rotating at 4°C for 15min. The amount of protein in the supernatant was determinate by the Bradford method using a Pierce Dilution-Free Rapid Gold BCA Protein Assay Kit (Thermo Fisher Scientific, Cat. No.: A55860) according to the supplier’s instructions. 0.5mg of protein in the supernatant were incubated with anti-CD56 antibody or isotype for 24 hours at 4°C. Then 30µL of lysate was incubated with Protein G Magnetic beads (New England Biolabs, Cat. No.: S1430S). Using a magnet (6-Tube Magnetic Separation Rack, Cell Signaling Technology), beads containing CD56 antibodies were isolated, the remaining lysate (supernatant) removed, and the beads were washed 5 times in RIPA lysis buffer with 1x Halt Protease Inhibitor (Thermo Fisher Scientific), and isolated. The remaining lysate, with CD56 removed, was also saved for analysis. Whole lysates were collected and stored until use at −20°C. For polySia digestion, T-cell lysates were processed as previously indicated and protein lysates were incubated for 1 hour at 37°C with 1μg of EndoN for 20μg of lysate. EndoN bacteriophage transcript sequence was used for plasmid generation and protein production was performed by GenScript.

### Immunoblotting

Naïve or stimulated T-cells were isolated from PBMCs and lysates were prepared as mentioned above. Cell lysates were resolved on a 4-12% bis-tris gradient Bis-Tris Plus WedgeWell polyacrylamide gel (Invitrogen, Cat. No.: 24020710) electrophoresis and transferred onto PVDF membrane (Millipore, Cat. no.: IPFL00010). Membranes were blocked in 5% skim milk for 1 hour at RT and incubated with the anti-polySia antibody in TBST overnight at 4°C. The following day, a fluorescent secondary anti-mouse antibody was added to the membranes for 1 hour at room temperature (RT) in the dark. The membranes were washed and then dried for 20 minutes and then visualized using a Sapphire Imager.

### Quantitative real-time PCR

Total RNA was isolated from naïve and stimulated T-cells according to the manufacture’s protocol (GENEzol TriRNA Pure Kit, Cat. No.: GZXD100). Pre-designed, gene-specific TaqMan^®^ probe and primer sets for ST8SIA4 (ThermoFisher Scientific, Cat. no.: Hs00379924) and NCAM1/CD56 (ThermoFisher Scientific, Cat. No.: Hs00941830_m1 ThermoFisher Scientific) were used for gene expression analysis. The relative expression level of each gene was normalized to an endogenous control, TBP gene (ThermoFisher Scientific, Cat. no.: Hs00427620_m1). The fold-differences were calculated using the ΔΔCT method, and the fold change was calculated as 2^(-ΔΔCT)^.

### Macrophage killing assay

Monocytes were differentiated into M1 macrophages as detailed above. Prior to co-culturing, a portion of macrophages cells were treated with 300ng/ml EndoN for 1 hour in a humidified, 5% CO2 incubator at 37°C. The MDA-MB-231 target cell line was stained with a cellular dye (Cell Trace Far Red) for 20 minutes in a humidified, 5% CO2 incubator at 37°C. Then the cells were washed in complete media and resuspended in IMDM (Corning) 10% human serum (Gemini). 100,000 target cells were added into each well containing macrophages. The cells were co-cultured for a 4-hour incubation in a humidified, 5% CO2 incubator at 37°C. After the incubation, the media was removed from the wells and the macrophage cells were washed off with a series of washes using PBS and enzyme-free dissociation solution (Millipore). The macrophages were stained with a macrophage-specific marker, anti-CD11b antibody for 40 minutes on ice in the dark. After incubation, the macrophages were lifted from the plate using TrypLE express (Gibco), and the samples were analyzed using flow cytometry. In each biological replicate, a minimum of two technical replicates were performed per experiment. The degree of phagocytosis was monitored by analyzing the median fluorescence intensity of macrophages that underwent phagocytosis. The median fluorescence intensity of cells positive for both CD11b+ and APC was normalized to the wildtype. The data compares between conditions, +/- EndoN, to identify if polySia plays a role in phagocytosis.

### Statistical analysis

The data was handled in excel and analyzed in GraphPad Prism (10.1.2). All data are expressed as mean ± SEM. Statistical analysis was conducted using GraphPad Prism. The data was analyzed for normality using the Shapiro-Wilk test, and depending on the normality results, either a one-way ANOVA test or a non-parametric Friedman test was conducted to determine significance. Where the p-value was determined to be <0.05, results were determined to be statistically significant.

## Data Availability

The raw data supporting the conclusions of this article will be made available by the authors, without undue reservation.
