# Supplementary material for: Polysialic acid is upregulated on activated immune cells and negatively regulates anticancer immune activity
Source: Front Oncol. 2025 Mar 20;15:1520948. doi: 10.3389/fonc.2025.1520948 (PMC11965634; doi:10.3389/fonc.2025.1520948)
Supplement: Supplementary file 1 [file DataSheet1.docx]

**SUPPLEMENTARY INFORMATION FOR**

**Polysialic acid is upregulated on activated immune cells and negatively regulates anticancer immune activity**

Olivia Drummond-Guy^1^, John Daly^1^, Angeline Wu^1^, Natalie Stewart^1^, Katy Milne^2^, Chloe Duff^2^, Brad H. Nelson^2,3,4^, Karla C. Williams^1*^, Simon Wisnovsky^1*^

^1^ University of British Columbia, Faculty of Pharmaceutical Sciences, Vancouver, BC.

^2^ Deeley Research Centre, BC Cancer, Victoria, BC V8R 6V5, Canada.

^3^ Department of Medical Genetics, University of British Columbia, Vancouver, BC V6T 1Z3, Canada.

^4^ Department of Biochemistry and Microbiology, University of Victoria, Victoria, BC V8P 3E6, Canada.

***** To whom correspondence should be addressed (karla.williams@ubc.ca & [simon.wisnovsky@ubc.ca](mailto:simon.wisnovsky@ubc.ca))


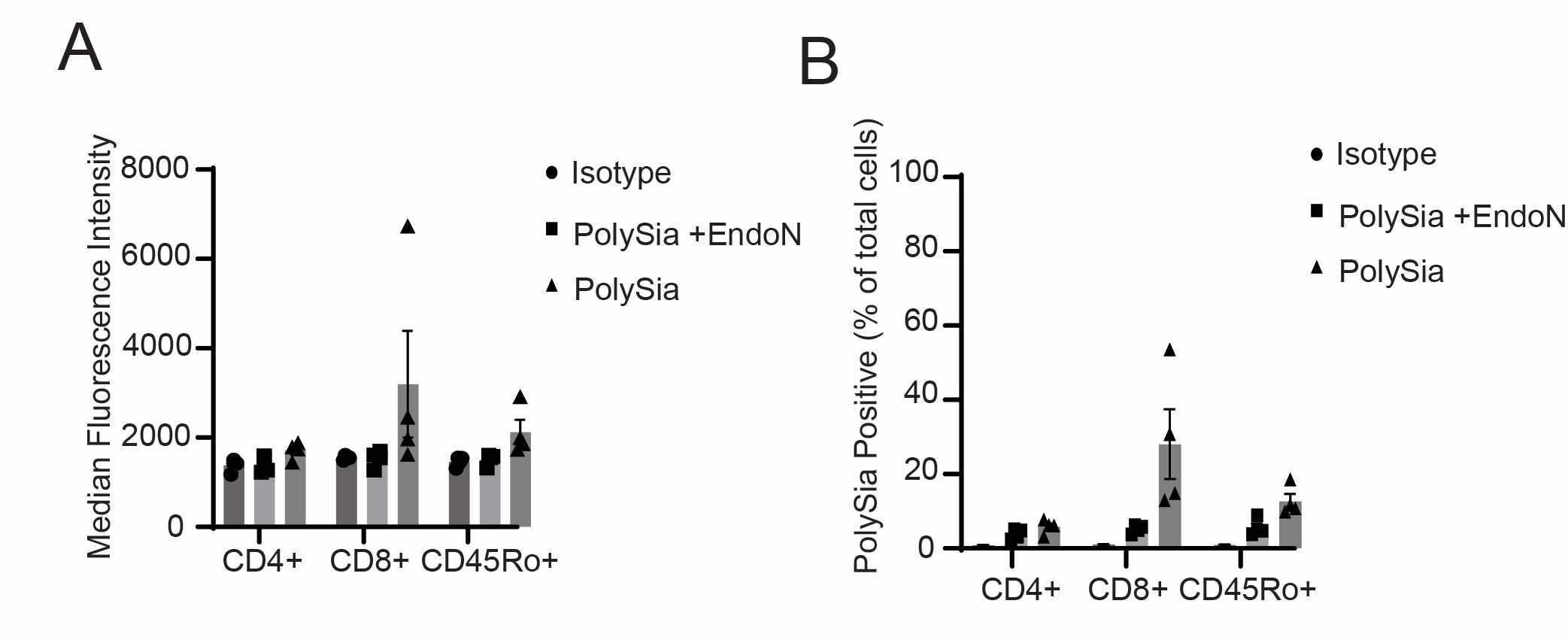


**Figure 1. Expression of PolySia on different T-cell subsets. A**) PBMCs were stained with either an anti-polySia antibody or an isotype control along with anti-CD3, CD4, CD8 and CD45RO antibodies. The median fluorescence intensity of polySia staining is shown for each indicated population. **B)** The percentage of polySia+ cells was gated based on the EndoN control and is shown for each indicated population. Mean values plotted, error bars indicate SEM.


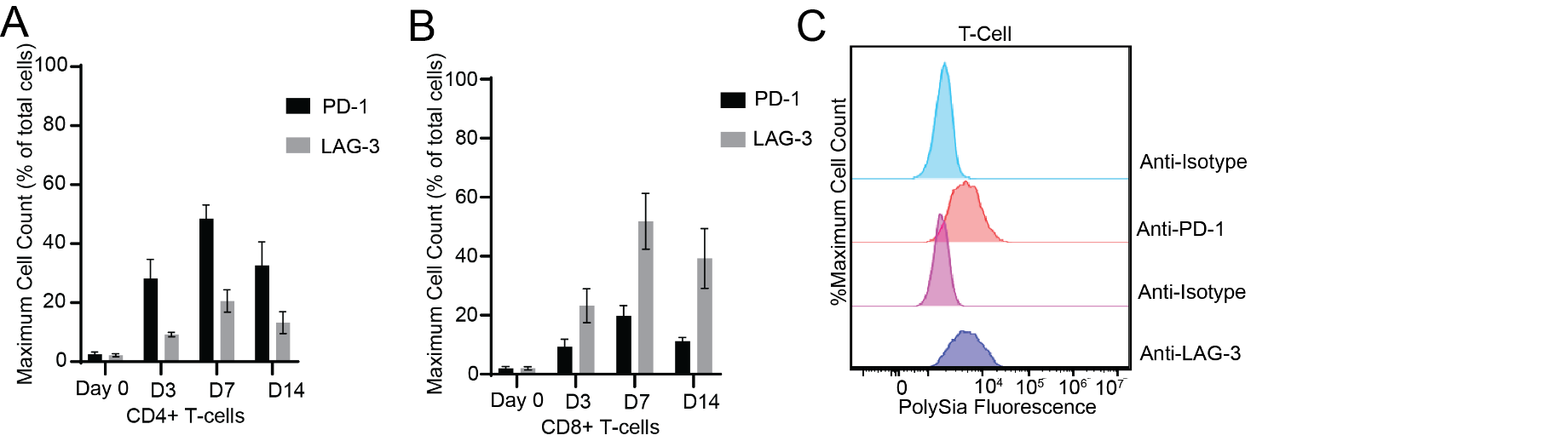


**Figure 2. Inhibitory checkpoint proteins are upregulated on stimulated T-cells. A)** T-cells were stimulated for 14 days as in **Fig. 3** and stained with an anti-PD-1, anti-LAG3 and anti-CD4 antibodies. The maximum cell counts of CD4+ T-cells positive for PD-1 and LAG-3 are plotted, n = 4 donors. **B)** T-cells were stimulated for 14 days and stained with an anti-PD-1, anti-LAG3 and anti-CD8 antibodies. The maximum cell counts of CD8+ T-cells positive for PD-1 and LAG-3 are plotted, n = 4 donors. **C)** The representative flow cytometry plot of T-cells stained with anti-PD-1, the anti-PD1 isotype, anti-LAG-3 and the anti-LAG-3 isotype antibodies 7 days after initial stimulation. Mean values plotted, error bars indicate SEM.

**
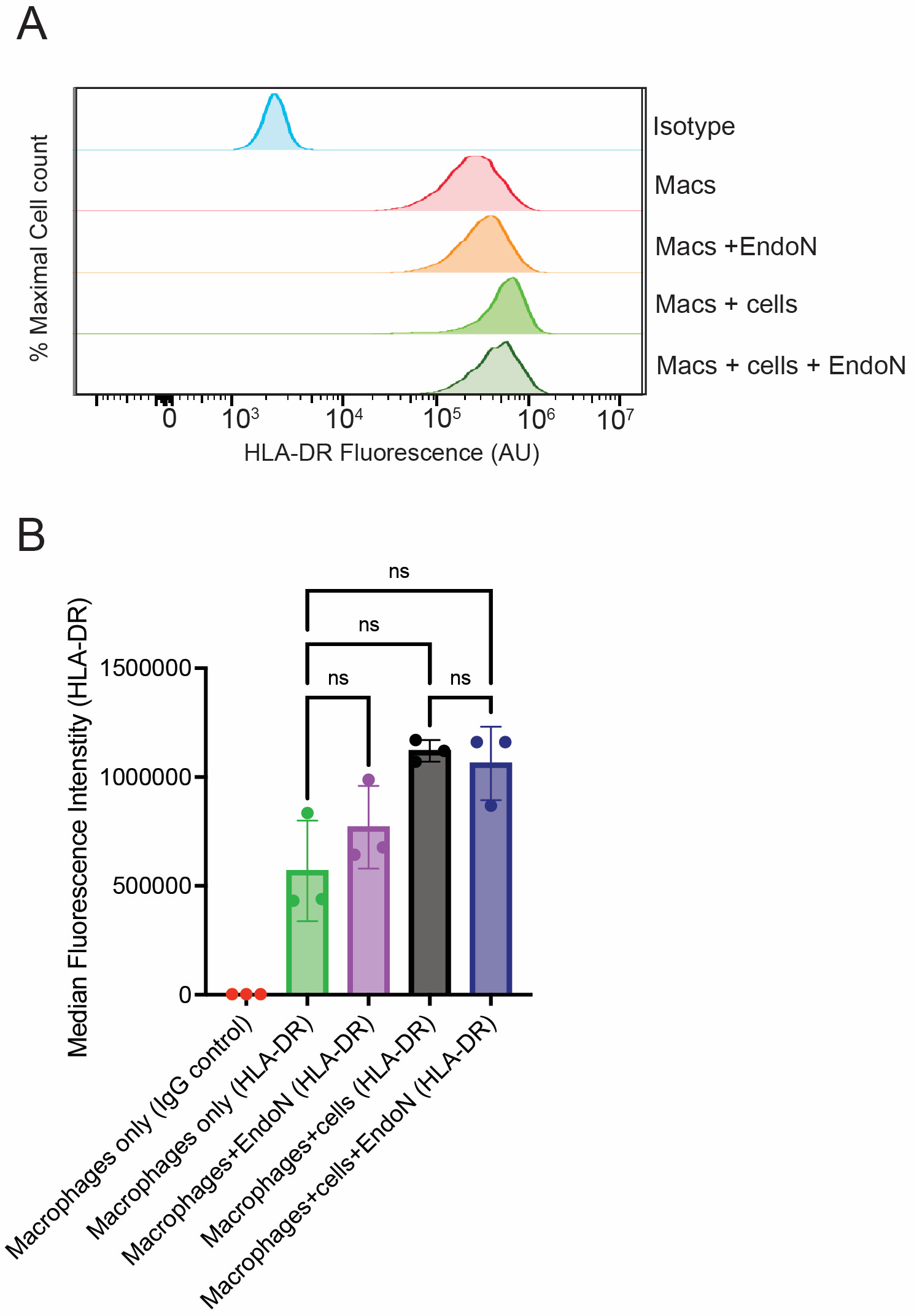
**

**Figure 3. MHC-II presentation in EndoN-treated macrophages. A**) Macrophages were differentiated, treated with EndoN and co-cultured with breast cancer cells. Four hours after incubation, macrophages were stained with anti-HLA-DR and CD11b antibodies. Representative flow cytometry plots of HLA-DR expression in CD11b+ cells are shown. B**)** The median fluorescence intensity of HLA-DR staining over n=3 independent replicates is shown. Error bars indicate SEM, statistical significance determined by Student’s two-tailed t-test.
